# Crystal structure of 3,14-diethyl-2,6,13,17-tetra­azoniatri­cyclo­[16.4.0.0^7,12^]docosane tetra­chloride tetra­hydrate from synchrotron X-ray data

**DOI:** 10.1107/S2056989021001006

**Published:** 2021-01-29

**Authors:** Dohyun Moon, Jong-Ha Choi

**Affiliations:** a Beamline Department, Pohang Accelerator Laboratory, POSTECH, Pohang 37673, Republic of Korea; b Department of Chemistry, Andong National University, Andong 36729, Republic of Korea

**Keywords:** crystal structure, tetra­protonated macrocycle, exodentate, tetra­chloride, hydrogen bonding, synchrotron radiation

## Abstract

The crystal structure of the hydrated title salt, C_22_H_48_N_4_^4+^·4Cl^−^·4H_2_O (C_22_H_48_N_4_ = H_4_ *L* = 3,14-diethyl-2,6,13,17-tetra­azoniatri­cyclo­[16.4.0.0^7,12^]doco­sa­ne), has been determined using synchrotron radiation at 220 K. The structure determination reveals that protonation has occurred at all four amine N atoms. The asymmetric unit comprises one half of the macrocyclic cation (completed by crystallographic inversion symmetry), two chloride anions and two water mol­ecules. The macrocyclic ring of the tetra­cation adopts an exodentate (3,4,3,4)-*D* conformation. The crystal structure is stabilized by inter­molecular hydrogen bonds involving the macrocycle N—H groups and water O—H groups as donors, and the O atoms of the water mol­ecules and chloride anions as acceptors, giving rise to a three-dimensional network.

## Abstract 2

In the hydrated title salt, [C_22_H_48_N_4_]Cl_4_·4H_2_O, the cation lies about an inversion centre. The macrocyclic ring adopts an exodentate (3,4,3,4)-*D* conformation. In the crystal, O—H⋯Cl, N—H⋯Cl and N—H⋯O hydrogen bonds connect the chloride anions, tetra­protonated cations and water mol­ecules into a three-dimensional network.

## Chemical context   

In recent years, derivatives of 1,4,8,11-tetra­aza­cyclo­tetra­decane (cyclam) have been found to exhibit anti-HIV effects (Ronconi & Sadler, 2007 ▸; Ross *et al.*, 2012 ▸) and to stimulate the activity of stem cells from bone marrow (De Clercq, 2010 ▸). The conformation of the macrocyclic ligand, the orientations of the N—H bonds and crystal packing forces in respective metal complexes are very important factors for CXCR4 chemokine receptor recognition. Therefore, knowledge of the conformations and crystal-packing features of complexes containing cyclam derivatives has become important in the development of new highly effective anti-HIV drugs that specifically target alternative events in the HIV replicative cycle. The macrocycle 3,14-diethyl-2,6,13,17-tetra­aza­tri­cyclo(16.4.0.07,12)docosane (C_22_H_44_N_4_, *L*) contains a cyclam backbone with two cyclo­hexane subunits. Ethyl groups are also attached to the 3 and 14 carbon atoms of the propyl chains that bridge opposite pairs of N atoms in the mol­ecule. The macrocycle *L* is a strongly basic amine capable of forming the dication C_22_H_46_N_4_^2+^ or even the tetra­cation C_22_H_48_N_4_^4+^ in which all of the N—H bonds are generally available for hydrogen-bond formation. It is known that the neutral macrocycle and its dication adopt an endodentate conformation along the centre of the macrocyclic cavity. The stabilization of such an endo conformation can be attributed to strong intra­molecular N—H⋯N hydrogen bonds. Unlike the free macrocycle and its dication, the tetra­cation adopts an exodentate conformation. Furthermore, the 14-membered cyclam moiety of the tetra­cation can adopt four exodentate (3,4,3,4)-(*A*–*D*) conformations (Meyer *et al.*, 1998 ▸; Nowicka *et al.*, 2012 ▸). Previously, the syntheses and crystal structures of the related compounds (C_22_H_44_N_4_)·NaClO_4_ (Aree *et al.*, 2018 ▸), [C_22_H_46_N_4_](ClO_4_)_2_ (Aree *et al.*, 2018 ▸), [C_22_H_46_N_4_]Cl_2_·4H_2_O (Moon *et al.*, 2013 ▸) and [C_22_H_46_N_4_](NO_3_)_2_·2H_2_O (Moon *et al.*, 2019 ▸) have been reported. However, there is no report of a compound with the 3,14-diethyl-2,6,13,17-tetra­azoniatri­cyclo­(16.4.0.0^7,12^)docosane cation and any counter-anions. As another contribution to our research on this macrocyclic compound family, we report here the preparation of a new tetra­cationic compound [C_22_H_48_N_4_]Cl_4_·4H_2_O, (I), as the hydrated tetra­chloride salt and its structural characterization by synchrotron single-crystal X-ray diffraction.
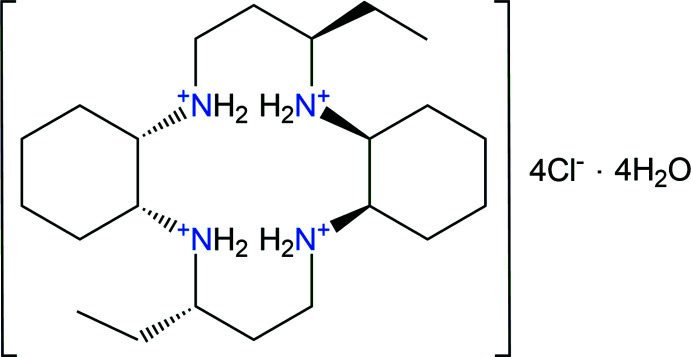


## Structural commentary   

**Figure 1:**
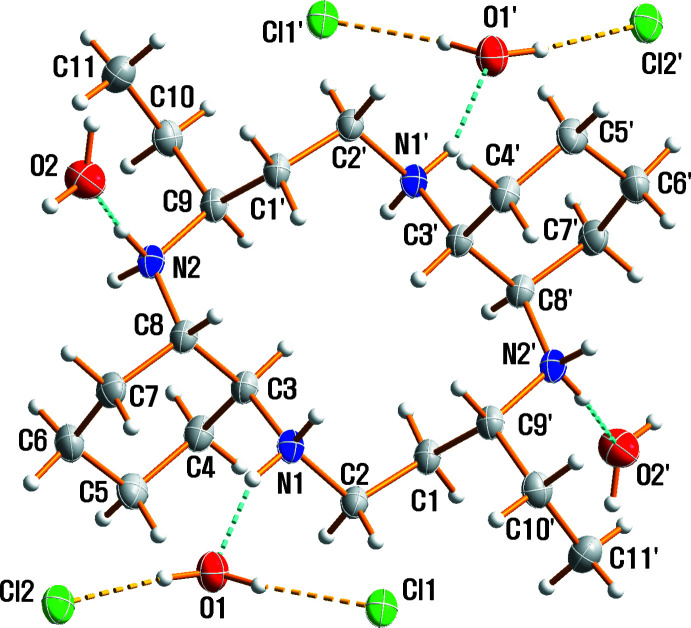
The mol­ecular structure of (I), drawn with displacement ellipsoids at the 50% probability level. Dashed lines represent hydrogen-bonding inter­actions; primed atoms are related by the symmetry operation (−*x* + 1, −*y* + 1, −*z* + 1).

The mol­ecular structure of (I) is shown in Fig. 1 ▸ along with the atom-numbering scheme. The organic cation lies across a crystallographic inversion centre and hence the asymmetric unit consists of one half of the cationic macrocycle, of two chloride anions and two solvent water mol­ecules. Within the centrosymmetric tetra­protonated amine unit C_22_H_48_N_4_^4+^, the C—C and N—C bond lengths range from 1.5208 (19) to 1.5431 (16) Å and from 1.5076 (15) to 1.5247 (15) Å, respectively; the range of N—C—C and C—N—C angles is 107.08 (9) to 111.72 (10)° and 116.40 (9) to 117.87 (9)°, respectively.

The four N atoms of the macrocycle are coplanar, and the two ethyl substituents are *anti* with respect to the macrocyclic plane as a result of the mol­ecular inversion symmetry. The six-membered cyclo­hexane ring is in its stable chair conformation. The cyclam moiety of the tetra­cation adopts an exodentate rectangular (3,4,3,4)-*D* conformation, which differs from the endodentate conformation of the free macrocycle or the dication (Aree *et al.*, 2018 ▸; Moon *et al.*, 2013 ▸). Only two of the four nitro­gen atoms, N2 and N2′ [symmetry code: (’) −*x* + 1, −*y* + 1, −*z* + 1] are located at the corners of the macrocyclic square. The other two corner positions are occupied by carbon atoms C2 and C2′. Thus, the remaining two nitro­gen atoms, N1 and N1′ are components of the hydro­carbon side chain. Inter­estingly, the *exo-*[3,4,3,4]-*D* conformation of (I) also differs from the *exo-*[3,4,3,4]-*B* conformation of [H_4_TMC](CrO_3_Cl)_2_Cl_2_ (TMC = 1,4,8,11-tetra­methyl-1,4,8,11-tetra­aza­cyclo­tetra­decane; Moon & Choi, 2020*a* ▸), and the *exo-*[3,4,3,4]-*C* conformation of [H_4_TMC](ClO_4_)_2_Cl_2_ (Moon & Choi, 2020*b* ▸) or (H_4_cyclam)[Cr_2_O_7_]_2_·H_2_O (Moon & Choi, 2017 ▸). The detailed understanding and insight into the crystal packing and conformation may be helpful in the development of new anti-HIV drugs.

## Supra­molecular features   

**Figure 2:**
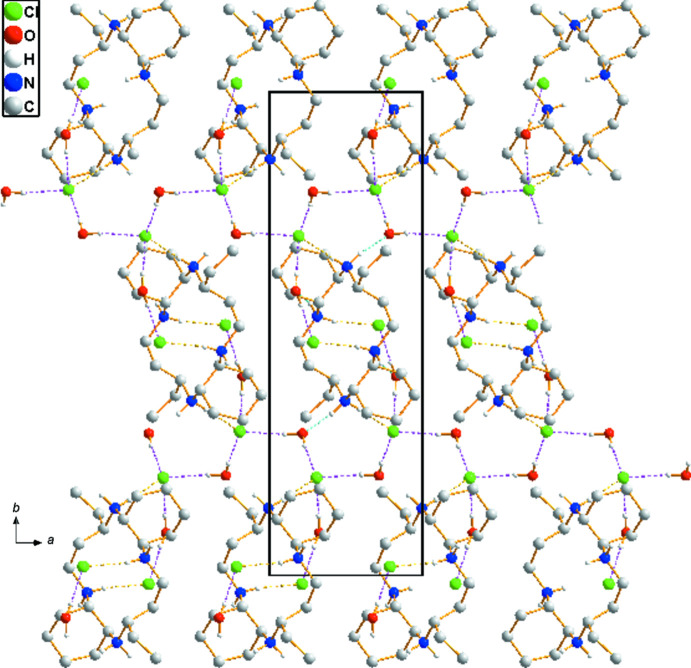
The crystal packing in (I), viewed perpendicular to the *ab* plane. Dashed lines represent O—H⋯Cl (pink), N—H⋯O (cyan), and N—H⋯Cl (yellow) hydrogen-bonding inter­actions, respectively. For clarity, C-bound H atoms have been omitted.

Extensive O—H⋯Cl, N—H⋯Cl and N—H⋯O hydrogen-bonding inter­actions occur in the crystal structure (Table 1 ▸). All of the chloride anions and the O atoms of the water mol­ecules serve as hydrogen-bond acceptors. The organic C_22_H_48_N_4_^4+^ cation is linked to four water mol­ecules *via* N—H⋯O hydrogen bonds whereas the O—H⋯Cl hydrogen bonds link the chloride anions to neighbouring water mol­ecules. In addition, neighbouring organic cations are inter­connected to chloride anions *via* several N—H⋯Cl hydrogen bonds. An extensive array of these contacts generates a three-dimensional network of mol­ecules. The crystal packing of (I) viewed perpendicular to the *ab* plane is shown in Fig. 2 ▸.

## Database survey   

A search of the Cambridge Structural Database (CSD; version 5.42, November 2020; Groom *et al.*, 2016 ▸) revealed five matches for organic compounds containing the macrocycles (C_22_H_44_N_4_), C_22_H_46_N_4_^2+^ or C_22_H_48_N_4_^4+^. The crystal structures of (C_22_H_44_N_4_)·NaClO_4_ (Aree *et al.*, 2018 ▸), [C_22_H_46_N_4_](ClO_4_)_2_ (Aree *et al.*, 2018 ▸), [C_22_H_46_N_4_]Cl_2_·4H_2_O (Moon *et al.*, 2013 ▸) and [C_22_H_46_N_4_](NO_3_)_2_·2H_2_O (Moon *et al.*, 2019 ▸) have been reported previously. All bond lengths and angles within the tetra­cation C_22_H_48_N_4_^4+^ in (I) are similar to those found in the database structures.

Until now, no crystal structure of a compound with the tetra­cation C_22_H_48_N_4_^4+^ and any counter-anion has been deposited.

## Synthesis and crystallization   

Ethyl vinyl ketone (97%), *trans*-1,2-cyclo­hexa­nedi­amine (99%) and copper(II) chloride dihydrate (99%) were purchased from Sigma–Aldrich and were used as received. All other chemicals were of analytical reagent grade. The solvents were of reagent grade and purified by usual methods. As a starting material, the 3,14-diethyl-2,6,13,17-tetra­aza­tri­cyclo(16.4.0.0^7,12^)docosane macrocycle *L* was prepared according to a published procedure (Lim *et al.*, 2006 ▸). A solution of *L* (0.091 g, 0.25 mmol) in water (10 mL) was added dropwise to a stirred solution of CuCl_2_·2H_2_O (0.085 g, 0.5 mmol) in water (15 mL). The solution was heated for 1 h at 373 K. After cooling to 298 K, the pH was adjusted to 3.0 by 1.0 *M* HCl. The solution was filtered and left at room temperature. A mixture of colourless, red and violet crystals formed from the solution over the next few days. The product mixture was added to a 30 ml MeOH–acetone (1:2 *v*:*v*) solution under stirring, and the stirring was continued for 30 min at 298 K. The red and violet compounds were manually removed, and block-like colorless single crystals of (I) suitable for X-ray analysis were obtained by filtration.

## Refinement   

Crystal data, data collection and structure refinement details are summarized in Table 2 ▸. All C- and N-bound H atoms in the complex were placed in geometrically idealized positions and constrained to ride on their parent atoms, with C—H distances of 0.97–0.99 Å, and with an N—H distance of 0.90 Å with *U*_iso_(H) values of 1.2 and 1.5*U*_eq_ of the parent atoms, respectively. O-bound H atoms of the water mol­ecules were located in a difference-Fourier map, and the O—H distances and the H—O—H angles were restrained using DFIX and DANG constraints (0.94 and 1.55 Å, respectively).

## Supplementary Material

Crystal structure: contains datablock(s) I. DOI: 10.1107/S2056989021001006/wm5597sup1.cif

Structure factors: contains datablock(s) I. DOI: 10.1107/S2056989021001006/wm5597Isup2.hkl

Supporting information file. DOI: 10.1107/S2056989021001006/wm5597Isup3.cml

CCDC reference: 2059324

Additional supporting information:  crystallographic information; 3D view; checkCIF report

## Figures and Tables

**Table 1:** Hydrogen-bond geometry (Å, °)

*D*—H⋯*A*	*D*—H	H⋯*A*	*D*⋯*A*	*D*—H⋯*A*
O1—H1*O*1⋯Cl2	0.92 (1)	2.25 (1)	3.1616 (11)	172 (1)
O1—H2*O*1⋯Cl1	0.92 (1)	2.17 (1)	3.0746 (11)	169 (1)
O2—H1*O*2⋯Cl2^i^	0.92 (1)	2.34 (1)	3.2518 (15)	172 (2)
O2—H2*O*2⋯Cl2^ii^	0.91 (1)	2.26 (1)	3.1622 (12)	173 (2)
N1—H1*A*⋯Cl1^iii^	0.90	2.22	3.1072 (12)	169
N1—H1*B*⋯O1	0.90	1.92	2.7740 (15)	157
N2—H2*A*⋯O2	0.90	1.91	2.7716 (14)	161
N2—H2*B*⋯Cl2^iv^	0.90	2.45	3.3357 (12)	167

Symmetry codes: (i) 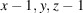; (ii) 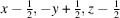; (iii) 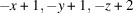; (iv) 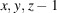.

**Table 2:** Experimental details

Crystal data	
Chemical formula	C_22_H_48_N_4_^4+^·4Cl^−^·4H_2_O
*M*_r_	582.50
Crystal system, space group	Monoclinic, *P*2_1_/*n*
Temperature (K)	220
*a*, *b*, *c* (Å)	7.6550 (15), 23.533 (5), 8.3130 (17)
β (°)	102.45 (3)
*V* (Å^3^)	1462.3 (5)
*Z*	2
Radiation type	Synchrotron, λ = 0.610 Å
μ (mm^−1^)	0.29
Crystal size (mm)	0.08 × 0.07 × 0.04

Data collection	
Diffractometer	Rayonix MX225HS CCD area detector
Absorption correction	Empirical (using intensity measurements) (*HKL3000sm *SCALEPACK**; Otwinowski *et al.*, 2003 ▸)
*T*_min_, *T*_max_	0.868, 1.000
No. of measured, independent and observed [*I* > 2σ(*I*)] reflections	14961, 4016, 3517
*R*_int_	0.038
(sin θ/λ)_max_ (Å^−1^)	0.693

Refinement	
*R*[*F* ^2^ > 2σ(*F* ^2^)], *wR*(*F* ^2^), *S*	0.035, 0.105, 1.09
No. of reflections	4016
No. of parameters	167
No. of restraints	6
H-atom treatment	H atoms treated by a mixture of independent and constrained refinement
Δρ_max_, Δρ_min_ (e Å^−3^)	0.41, −0.22

Computer programs: *PAL BL2D-SMDC* (Shin *et al.*, 2016 ▸), *HKL3000sm* (Otwinowski *et al.*, 2003 ▸), *SHELXT2018* (Sheldrick, 2015*a* ▸), *SHELXL2018* (Sheldrick, 2015*b* ▸), *DIAMOND* (Putz & Brandenburg, 2014 ▸) and *publCIF* (Westrip, 2010 ▸).

## supplementary crystallographic information

### Crystal data

**Table:**

C_22_H_48_N_4_^4+^·4Cl^−^·4H_2_O	*F*(000) = 632
*M**_r_* = 582.50	*D*_x_ = 1.323 Mg m^−^^3^
Monoclinic, *P*2_1_/*n*	Synchrotron radiation, λ = 0.610 Å
*a* = 7.6550 (15) Å	Cell parameters from 50732 reflections
*b* = 23.533 (5) Å	θ = 0.4–33.7°
*c* = 8.3130 (17) Å	µ = 0.28 mm^−^^1^
β = 102.45 (3)°	*T* = 220 K
*V* = 1462.3 (5) Å^3^	Block, colorless
*Z* = 2	0.08 × 0.07 × 0.04 mm

### Data collection

**Table:**

Rayonix MX225HS CCD area detector diffractometer	3517 reflections with *I* > 2σ(*I*)
Radiation source: PLSII 2D bending magnet	*R*_int_ = 0.038
ω scan	θ_max_ = 25.0°, θ_min_ = 1.5°
Absorption correction: empirical (using intensity measurements) (*HKL3000sm Scalepack*; Otwinowski *et al.*, 2003)	*h* = −10→10
*T*_min_ = 0.868, *T*_max_ = 1.000	*k* = −32→32
14961 measured reflections	*l* = −11→11
4016 independent reflections	

### Refinement

**Table:**

Refinement on *F*^2^	6 restraints
Least-squares matrix: full	Hydrogen site location: mixed
*R*[*F*^2^ > 2σ(*F*^2^)] = 0.035	H atoms treated by a mixture of independent and constrained refinement
*wR*(*F*^2^) = 0.105	*w* = 1/[σ^2^(*F*_o_^2^) + (0.0597*P*)^2^ + 0.2081*P*] where *P* = (*F*_o_^2^ + 2*F*_c_^2^)/3
*S* = 1.09	(Δ/σ)_max_ = 0.001
4016 reflections	Δρ_max_ = 0.41 e Å^−^^3^
167 parameters	Δρ_min_ = −0.22 e Å^−^^3^

### Special details

**Table:**

Geometry. All esds (except the esd in the dihedral angle between two l.s. planes) are estimated using the full covariance matrix. The cell esds are taken into account individually in the estimation of esds in distances, angles and torsion angles; correlations between esds in cell parameters are only used when they are defined by crystal symmetry. An approximate (isotropic) treatment of cell esds is used for estimating esds involving l.s. planes.

### Fractional atomic coordinates and isotropic or equivalent isotropic displacement parameters (Å^2^)

**Table:**

	*x*	*y*	*z*	*U*_iso_*/*U*_eq_	
Cl1	0.71371 (4)	0.51779 (2)	1.23367 (4)	0.02947 (10)	
Cl2	0.80805 (4)	0.29797 (2)	1.26093 (4)	0.03510 (10)	
O1	0.82713 (14)	0.41203 (4)	1.06447 (12)	0.0341 (2)	
H1O1	0.822 (2)	0.3768 (4)	1.1119 (19)	0.041*	
H2O1	0.792 (2)	0.4405 (5)	1.1271 (18)	0.041*	
O2	0.21622 (14)	0.29355 (5)	0.47672 (14)	0.0405 (2)	
H1O2	0.1034 (16)	0.2916 (8)	0.4106 (19)	0.049*	
H2O2	0.233 (2)	0.2681 (6)	0.5609 (16)	0.049*	
N1	0.68792 (13)	0.46398 (4)	0.76471 (12)	0.0234 (2)	
H1A	0.576535	0.473733	0.773054	0.028*	
H1B	0.728900	0.438497	0.844668	0.028*	
N2	0.49393 (13)	0.36101 (4)	0.42080 (12)	0.0239 (2)	
H2A	0.409339	0.334408	0.420241	0.029*	
H2B	0.588974	0.343395	0.395724	0.029*	
C1	0.74209 (15)	0.56567 (5)	0.68089 (15)	0.0247 (2)	
H1C	0.713036	0.551403	0.567493	0.030*	
H1D	0.840569	0.592881	0.689320	0.030*	
C2	0.80442 (16)	0.51617 (5)	0.79715 (16)	0.0255 (2)	
H2C	0.926151	0.505782	0.788904	0.031*	
H2D	0.809475	0.528837	0.910366	0.031*	
C3	0.67631 (15)	0.43519 (5)	0.60043 (14)	0.0232 (2)	
H3	0.622610	0.462009	0.511758	0.028*	
C4	0.86023 (16)	0.41738 (5)	0.57551 (15)	0.0270 (2)	
H4A	0.848164	0.401294	0.464927	0.032*	
H4B	0.937435	0.450940	0.583476	0.032*	
C5	0.94712 (16)	0.37373 (6)	0.70283 (17)	0.0307 (3)	
H5A	0.968765	0.390841	0.812822	0.037*	
H5B	1.062841	0.362317	0.680786	0.037*	
C6	0.82840 (18)	0.32133 (6)	0.69882 (17)	0.0325 (3)	
H6A	0.881223	0.296219	0.790489	0.039*	
H6B	0.825026	0.300529	0.596063	0.039*	
C7	0.63628 (17)	0.33618 (5)	0.71111 (16)	0.0288 (3)	
H7A	0.562168	0.301986	0.687145	0.035*	
H7B	0.636910	0.347551	0.824645	0.035*	
C8	0.55016 (15)	0.38374 (5)	0.59487 (14)	0.0237 (2)	
H8	0.441439	0.396678	0.630583	0.028*	
C9	0.42198 (15)	0.40325 (5)	0.28341 (14)	0.0240 (2)	
H9	0.516285	0.431990	0.282803	0.029*	
C10	0.38798 (17)	0.37228 (5)	0.11758 (15)	0.0285 (2)	
H10A	0.361097	0.400848	0.029936	0.034*	
H10B	0.499099	0.353289	0.107674	0.034*	
C11	0.2386 (2)	0.32840 (6)	0.08671 (18)	0.0371 (3)	
H11A	0.259808	0.300603	0.174946	0.056*	
H11B	0.235881	0.309471	−0.017459	0.056*	
H11C	0.124952	0.347133	0.082958	0.056*	

### Atomic displacement parameters (Å^2^)

**Table:**

	*U*^11^	*U*^22^	*U*^33^	*U*^12^	*U*^13^	*U*^23^
Cl1	0.02435 (15)	0.02771 (17)	0.04097 (18)	−0.00112 (10)	0.01724 (12)	−0.00061 (11)
Cl2	0.03364 (17)	0.03180 (18)	0.04254 (19)	0.00500 (12)	0.01410 (14)	−0.00293 (12)
O1	0.0398 (5)	0.0299 (5)	0.0361 (5)	0.0030 (4)	0.0156 (4)	0.0005 (4)
O2	0.0314 (5)	0.0430 (6)	0.0482 (6)	−0.0074 (4)	0.0111 (4)	0.0115 (5)
N1	0.0220 (4)	0.0202 (5)	0.0305 (5)	0.0007 (3)	0.0112 (4)	0.0001 (4)
N2	0.0223 (4)	0.0193 (4)	0.0322 (5)	−0.0009 (3)	0.0107 (4)	−0.0002 (4)
C1	0.0242 (5)	0.0191 (5)	0.0339 (6)	−0.0003 (4)	0.0134 (4)	−0.0003 (4)
C2	0.0227 (5)	0.0204 (5)	0.0343 (6)	−0.0008 (4)	0.0083 (4)	−0.0021 (4)
C3	0.0229 (5)	0.0199 (5)	0.0290 (5)	−0.0017 (4)	0.0106 (4)	−0.0011 (4)
C4	0.0250 (5)	0.0241 (5)	0.0361 (6)	−0.0028 (4)	0.0161 (5)	−0.0031 (5)
C5	0.0240 (5)	0.0294 (6)	0.0410 (6)	0.0034 (5)	0.0123 (5)	−0.0023 (5)
C6	0.0343 (6)	0.0239 (6)	0.0400 (7)	0.0035 (5)	0.0097 (5)	0.0025 (5)
C7	0.0299 (6)	0.0224 (6)	0.0352 (6)	−0.0038 (4)	0.0096 (5)	0.0036 (5)
C8	0.0224 (5)	0.0210 (5)	0.0304 (5)	−0.0019 (4)	0.0114 (4)	−0.0011 (4)
C9	0.0229 (5)	0.0203 (5)	0.0312 (5)	−0.0006 (4)	0.0114 (4)	0.0017 (4)
C10	0.0292 (6)	0.0284 (6)	0.0310 (6)	0.0044 (5)	0.0133 (5)	−0.0006 (5)
C11	0.0415 (7)	0.0312 (7)	0.0381 (7)	−0.0035 (6)	0.0077 (6)	−0.0063 (6)

### Geometric parameters (Å, º)

**Table:**

O1—H1O1	0.924 (9)	C4—C5	1.5213 (18)
O1—H2O1	0.922 (9)	C4—H4A	0.9800
O2—H1O2	0.919 (9)	C4—H4B	0.9800
O2—H2O2	0.910 (9)	C5—C6	1.5279 (19)
N1—C2	1.5076 (15)	C5—H5A	0.9800
N1—C3	1.5099 (15)	C5—H5B	0.9800
N1—H1A	0.9000	C6—C7	1.5364 (18)
N1—H1B	0.9000	C6—H6A	0.9800
N2—C8	1.5155 (16)	C6—H6B	0.9800
N2—C9	1.5247 (15)	C7—C8	1.5318 (17)
N2—H2A	0.9000	C7—H7A	0.9800
N2—H2B	0.9000	C7—H7B	0.9800
C1—C2	1.5231 (17)	C8—H8	0.9900
C1—C9^i^	1.5366 (16)	C9—C10	1.5312 (17)
C1—H1C	0.9800	C9—H9	0.9900
C1—H1D	0.9800	C10—C11	1.5208 (19)
C2—H2C	0.9800	C10—H10A	0.9800
C2—H2D	0.9800	C10—H10B	0.9800
C3—C4	1.5253 (16)	C11—H11A	0.9700
C3—C8	1.5431 (16)	C11—H11B	0.9700
C3—H3	0.9900	C11—H11C	0.9700
			
H1O1—O1—H2O1	111.5 (13)	C6—C5—H5A	109.4
H1O2—O2—H2O2	112.6 (15)	C4—C5—H5B	109.4
C2—N1—C3	116.40 (9)	C6—C5—H5B	109.4
C2—N1—H1A	108.2	H5A—C5—H5B	108.0
C3—N1—H1A	108.2	C5—C6—C7	112.85 (11)
C2—N1—H1B	108.2	C5—C6—H6A	109.0
C3—N1—H1B	108.2	C7—C6—H6A	109.0
H1A—N1—H1B	107.3	C5—C6—H6B	109.0
C8—N2—C9	117.87 (9)	C7—C6—H6B	109.0
C8—N2—H2A	107.8	H6A—C6—H6B	107.8
C9—N2—H2A	107.8	C8—C7—C6	114.35 (10)
C8—N2—H2B	107.8	C8—C7—H7A	108.7
C9—N2—H2B	107.8	C6—C7—H7A	108.7
H2A—N2—H2B	107.2	C8—C7—H7B	108.7
C2—C1—C9^i^	113.50 (10)	C6—C7—H7B	108.7
C2—C1—H1C	108.9	H7A—C7—H7B	107.6
C9^i^—C1—H1C	108.9	N2—C8—C7	109.85 (10)
C2—C1—H1D	108.9	N2—C8—C3	110.65 (9)
C9^i^—C1—H1D	108.9	C7—C8—C3	111.90 (10)
H1C—C1—H1D	107.7	N2—C8—H8	108.1
N1—C2—C1	114.66 (10)	C7—C8—H8	108.1
N1—C2—H2C	108.6	C3—C8—H8	108.1
C1—C2—H2C	108.6	N2—C9—C10	109.14 (9)
N1—C2—H2D	108.6	N2—C9—C1^i^	110.12 (9)
C1—C2—H2D	108.6	C10—C9—C1^i^	114.39 (10)
H2C—C2—H2D	107.6	N2—C9—H9	107.6
N1—C3—C4	111.72 (10)	C10—C9—H9	107.6
N1—C3—C8	107.08 (9)	C1^i^—C9—H9	107.6
C4—C3—C8	111.74 (10)	C11—C10—C9	116.80 (10)
N1—C3—H3	108.7	C11—C10—H10A	108.1
C4—C3—H3	108.7	C9—C10—H10A	108.1
C8—C3—H3	108.7	C11—C10—H10B	108.1
C5—C4—C3	111.65 (10)	C9—C10—H10B	108.1
C5—C4—H4A	109.3	H10A—C10—H10B	107.3
C3—C4—H4A	109.3	C10—C11—H11A	109.5
C5—C4—H4B	109.3	C10—C11—H11B	109.5
C3—C4—H4B	109.3	H11A—C11—H11B	109.5
H4A—C4—H4B	108.0	C10—C11—H11C	109.5
C4—C5—C6	111.17 (11)	H11A—C11—H11C	109.5
C4—C5—H5A	109.4	H11B—C11—H11C	109.5
			
C3—N1—C2—C1	63.32 (13)	C6—C7—C8—N2	76.17 (13)
C9^i^—C1—C2—N1	74.65 (13)	C6—C7—C8—C3	−47.14 (14)
C2—N1—C3—C4	57.74 (13)	N1—C3—C8—N2	165.93 (9)
C2—N1—C3—C8	−179.62 (9)	C4—C3—C8—N2	−71.44 (12)
N1—C3—C4—C5	62.83 (13)	N1—C3—C8—C7	−71.22 (12)
C8—C3—C4—C5	−57.11 (13)	C4—C3—C8—C7	51.41 (13)
C3—C4—C5—C6	57.11 (13)	C8—N2—C9—C10	175.45 (9)
C4—C5—C6—C7	−52.02 (14)	C8—N2—C9—C1^i^	−58.20 (12)
C5—C6—C7—C8	47.89 (15)	N2—C9—C10—C11	67.17 (13)
C9—N2—C8—C7	−174.08 (9)	C1^i^—C9—C10—C11	−56.69 (15)
C9—N2—C8—C3	−50.04 (12)		

Symmetry code: (i) −*x*+1, −*y*+1, −*z*+1.

### Hydrogen-bond geometry (Å, º)

**Table:**

*D*—H···*A*	*D*—H	H···*A*	*D*···*A*	*D*—H···*A*
O1—H1*O*1···Cl2	0.92 (1)	2.25 (1)	3.1616 (11)	172 (1)
O1—H2*O*1···Cl1	0.92 (1)	2.17 (1)	3.0746 (11)	169 (1)
O2—H1*O*2···Cl2^ii^	0.92 (1)	2.34 (1)	3.2518 (15)	172 (2)
O2—H2*O*2···Cl2^iii^	0.91 (1)	2.26 (1)	3.1622 (12)	173 (2)
N1—H1*A*···Cl1^iv^	0.90	2.22	3.1072 (12)	169
N1—H1*B*···O1	0.90	1.92	2.7740 (15)	157
N2—H2*A*···O2	0.90	1.91	2.7716 (14)	161
N2—H2*B*···Cl2^v^	0.90	2.45	3.3357 (12)	167

Symmetry codes: (ii) *x*−1, *y*, *z*−1; (iii) *x*−1/2, −*y*+1/2, *z*−1/2; (iv) −*x*+1, −*y*+1, −*z*+2; (v) *x*, *y*, *z*−1.
